# Mechanisms by which silencing long-stranded noncoding RNA KCNQ1OT1 alleviates myocardial ischemia/reperfusion injury (MI/RI)-induced cardiac injury via miR-377-3p/HMOX1

**DOI:** 10.1186/s12872-023-03693-y

**Published:** 2024-01-03

**Authors:** Tongcai Tan, Liang Tu, Yanmei Yu, MinJie He, Xingchao Zhou, Lei Yang

**Affiliations:** 1 Center for Rehabilitation Medicine, Rehabilitation & Sports Medicine Research Institute of Zhejiang Province, Department of Rehabilitation Medicine, Zhejiang Provincial People’s Hospital (Affiliated People’s Hospital, Hangzhou Medical College, Hangzhou, Zhejiang 310014 China; 2 Medical Experimental Center, The First Affiliated Hospital of Chongqing Medical and Pharmaceutical College (The 6th People Hospital of Chongqing), Chongqing, 400060 China; 3 Geriatric Department, First Affiliated Hospital of Kunming Medical University, Kunming, Yunnan 650000 China; 4 Department of Medical Equipment, The First Affiliated Hospital of Dali University, Dali, Yunnan 671000 China; 5 Department of Rehabilitation Medicine, The Second People’s Hospital of Kunming, Kunming, Yunnan 650506 China

**Keywords:** Myocardial ischemia-reperfusion injury, lncRNA KCNQ1OT1, miR-377-3p, HMOX1, Oxidative stress

## Abstract

**Background:**

The key complication of myocardial infarction therapy is myocardial ischemia/reperfusion injury (MI/RI), and there is no effective treatment. The present study elucidates the mechanism of action of lncRNA KCNQ1OT1 in alleviating MI/RI and provides new perspectives and therapeutic targets for cardiac injury-related diseases.

**Methods:**

An ischemia/reperfusion (I/R) injury model of human adult cardiac myocytes (HACMs) was constructed, and the expression of KCNQ1OT1 and miR-377-3p was determined by RT‒qPCR. The levels of related proteins were detected by western blot analysis. Cell proliferation was detected by a CCK-8 assay, and cell apoptosis and ROS content were determined by flow cytometry. SOD and MDA expression as well as Fe^2+^ changes were detected by related analysis kits. The target binding relationships between lncRNA KCNQ1OT1 and miR-377-3p as well as between miR-377-3p and heme oxygenase 1 (HMOX1) were verified by a dual-luciferase reporter gene assay.

**Results:**

Myocardial ischemia‒reperfusion caused oxidative stress in HACMs, resulting in elevated ROS levels, increased Fe^2+^ levels, decreased cell viability, and increased LDH release (a marker of myocardial injury), and apoptosis. KCNQ1OT1 and HMOX1 were upregulated in I/R-induced myocardial injury, but the level of miR-377-3p was decreased. A dual-luciferase reporter gene assay indicated that lncRNA KCNQ1OT1 targets miR-377-3p and that miR-377-3p targets HMOX1. Inhibition of HMOX1 alleviated miR-377-3p downregulation-induced myocardial injury. Furthermore, lncRNA KCNQ1OT1 promoted the level of HMOX1 by binding to miR-377-3p and aggravated myocardial injury.

**Conclusion:**

LncRNA KCNQ1OT1 aggravates ischemia‒reperfusion-induced cardiac injury via miR-377-3P/HMOX1.

## Introduction

Myocardial ischemia‒reperfusion injury (MI/RI) is a key complication of reperfusion therapy for myocardial infarction [1]. Although reperfusion improves patient survival by rapidly restoring collateral flow in infarct-related coronary arteries, it itself triggers myocardial injury [2]. Although an increasing number of studies are dedicated to finding new targets to improve the clinical benefit of treatment for this disease, the gene expression and its regulatory mechanism during I/R injury are not completely clear [3].

Oxidative stress (OS) is defined as a disturbance in the pro-/antioxidant balance [4]. During I/R injury, oxidative stress plays a key role in the activation of iron death. Iron-driven cell death is characterized by iron accumulation and lipid peroxidation [5]. Heme oxygenase 1 (HMOX1) activation mediates the release of free iron ions from heme, and plays a key role in ferroptosis-induced ROS [6, 7]. In addition, it has been reported that some miRNAs have been designated regulators of oxidative stress in the cardiovascular system [8].

Long-stranded noncoding RNA (lncRNA) is a type of nonprotein-coding RNA that is more than 200 nucleotides in length, and lncRNA is involved in chromosome modification, genome modification, transcription inhibition, and activation of related genes in cells [9, 10]. It has been reported that reverse chain/antisense transcript 1 (KCNQ1OT1) of KCNQ1 is involved in the regulation of the cell cycle, invasion, proliferation, migration, glucose metabolism, and immune escape of cancer cells [11–13]. Downregulation of lncRNA KCNQ1OT1 protects against MI/RI following acute myocardial infarction [14, 15]. However, studies on lncRNA KCNQ1OT1 in MI/RI are scarce.

As a class of small noncoding RNAs with 18–25 nucleotides, microRNAs regulate the expression of related genes at the posttranscriptional level [16]. LncRNAs act as competitive endogenous RNAs (ceRNAs) to target related miRNAs, thereby affecting and regulating the expression of related genes [17]. The lncRNA‒miRNA-mRNA ceRNA network has been shown to have an integral role in I/R injury [18–20], and Yipeng Mo et al. [21] reported that lncRNA cardiac hypertrophy-related factor (CHRF) exacerbates myocardial I/R injury by regulating the miR-182-5p/ATG7 pathway to enhance autophagy. YongQuan Chen et al. [22] found that knocking out lncRNA TTTY15 alleviates MI/RI through the miR-374a-5p/FOXO1 axis. MiR-377-3p is closely related to the proliferation, apoptosis, migration, and inflammation of vascular smooth muscle cells (VSMCs) [23]. There is an unclear regulatory network between lncRNA KCNQ1OT1 and miR-377-3p in I/R injury.

This study aims to investigate the role and mechanism of lncRNA KCNQ1OT1, miRNA-377-3p and HMOX1 in MI/RI, as well as the way the genes are regulated.

## Experimental method

### Cell culture

Human adult cardiac myocytes (HACMs) were purchased form Otwo Biotech (Shenzhen, China), cultured in complete DMEM medium (10% fetal bovine serum and 1% streptomycin, Gibco, USA) at 5% CO_2_ and 37℃. HACMs were cultured in a hypoxic incubator with an atmosphere of 94% N_2_, 1% O_2_, and 5% CO_2_ for 4 h to simulate ischemia. Subsequently, cells were transferred to the aforementioned complete medium. Thus, the H/R model was successfully constructed.

### Cell transfection

The lncRNA KCNQ1OT1 overexpression vector (OE-KCNQ1OT1), siRNA against lncRNA KCNQ1OT1 (si-KCNQ1OT1), miR-377-3p inhibitor, miR-377-3p mimic, and siRNA against HMOX1 (si-HMOX1) were synthesized by Shanghai GenePharma Co., Ltd. (China). Transfection was performed using Lipofectamine 3000 (Invitrogen, USA) according to the manufacturer’s instructions, and cells were harvested after 48 h of culture.

### RT‒qPCR

Total RNA Extractor (Sangon Biotech) was used to extract total RNA from HACMs. A cDNA synthesis kit (Vazyme, Nanjing, China) was used to reverse transcribe 2 µg of RNA into cDNA, which was diluted 10-fold. The prepared cDNA (1 µl) was used for RT‒qPCR. β-actin and U6 were used as references. All primers (Table 1) used in this study were designed with Premier 5.0. The confidence of the PCR results was assessed by the dissociation curve and cycle threshold (CT) values. The results were calculated by the 2^−ΔΔCt^ method.

**Table 1:** Primer sequences (5’-3’)

Genes	Forward Primer	Reverse Primer
LncRNA KCNQ1OT1	CTTTGCAGCAACCTCCTTGT	TGGGGTGAGGGATCTGAA
hsa/mmu-miR-377-3p	CGCGATCACACAAAGGCAAC	AGTGCAGGGTCCGAGGTATT
Has-HMOX1	AAGACTGCGTTCCTGCTCAAC	AAAGCCCTACAGCAACTGTCG
mmu-HMOX1	AAGCCGAGAATGCTGAGTTCA	GCCGTGTAGATATGGTACAAGGA
hsa/mmu-U6	CTCGCTTCGGCAGCACA	AACGCTTCACGAATTTGCGT
hsa-β-actin	CATGTACGTTGCTATCCAGGC	CTCCTTAATGTCACGCACGAT
mmu-β-actin	GGCTGTATTCCCCTCCATCG	CCAGTTGGTAACAATGCCATGT

### Detection of apoptosis by flow cytometry

Flow cytometry was used to measure cell apoptosis. After treatment of cells, they were collected in a flow tube, centrifuged at 4 °C, washed with PBS, resuspended, and incubated with Annexin V-fluorescein isothiocyanate (FITC)/propidine iodide (PI). Annexin V-FITC and PI fluorescence was determined by a flow cytometer (BD Biosciences) and FlowJo software (V11).

### Reactive oxygen species (ROS) detection

After trypsin digestion, the cell density of each group of cells was adjusted with cell culture medium, and 2 × 10^5^ cells were incubated in 6-well plates in a cell culture incubator containing 5% CO_2_ for 24 h. After centrifugation, the medium was discarded, and cells were resuspended in 500 µL of D-Hanks Balanced Salt Solution containing DCFH-DA (20 µL) followed by incubation for 30 min (shaken every 5 min) in an incubator protected from light. Subsequently, cells were resuspended with D-Hanks Balanced Salt Solution, and flow cytometry was used to detect the levels of ROS.

In addition, mitochondrial superoxide production was determined by MitoSOX Red (M36008, MAOKANG, China). Cells were incubated with 5 μm MitoSOX^™^ in the dark at 37 ℃ for 30 min and imaged by laser scanning confocal at excitation wavelength 510 nm and emission wavelength 580 nm (LSM510, Zeiss, Germany).

### CCK-8

A CCK-8 assay was used to measure the proliferation of HACMs. After transfection, log-phase cells were collected, and the concentration of the cell suspension was adjusted by adding 100 µL per well to a 96-well plate (4,000 cells/well). Cells were cultured at 37 °C for 0, 24, and 48 h. After transfection or dosing, 10 µL of CCK-8 reagent was added followed by incubation for 2 h. Finally, an enzyme marker (ELX800, BioTeK, UK) was used to measure the absorbance at 450 nm. Each experiment was repeated 3 times independently.

### Dual-luciferase reporter gene

The 3′-UTR of KCNQ1OT1 was inserted into the pGL3 luciferase reporter vector (Promega, USA). Mutations were inserted into the seed region of the miR-377-3p-binding site of the 3′-UTR of KCNQ1OT1 by overlapping extension PCR. Cells were cotransfected with wild‐type pGL3‐KCNQ1OT1‐3′‐UTR or mutant KCNQ1OT1‐3′‐UTR and a scrambled miRNA control or miR‐29b‐3p mimics using Lipofectamine 2000 (Invitrogen, USA). After 48 h, luciferase activity was determined by the dual-luciferase reporter assay kit (Promega, USA) according to the manufacturer’s instructions. HMOX1 was determined by the same method as above.

### Fe^2+^ testing

Fe^2+^ was detected in HACMs using an iron assay kit according to the manufacturer’s instructions and previously described methods (Li et al., 2020).

### Western blot analysis

Proteins were extracted utilizing RIPA lysis buffer (Sangon Biotech, Shanghai) containing benzoyl fluoride (PMSF). A BCA assay (Sangon Biotech, Shanghai) was used to determine the total protein concentration. Protein samples (50 µg) were electrophoresed on SDS‒PAGE gels and then transferred to polyvinylidene fluoride membranes. The membranes were blocked with skim milk powder (5%) for 2 h followed by incubation overnight with the following primary antibodies (Abcam, UK): anti-CK (ab302638, 1:1000), anti-LDH (ab53292, 1:1000), anti-Bax (ab32503, 1:2000), anti-Bcl-2 (ab182858, 1:2000), and anti-GAPDH (ab8245, 1:1000). The membranes were then washed with TBST buffer and incubated with secondary antibodies at 25 °C for 1 h. Subsequently, chemiluminescent reagents were added, and the bands were analyzed for grayscale values using ImageJ software.

### Oxidative stress factor (SOD and MDA) assay

HACMs were lysed with RIPA buffer (Beyotime, China), and the cell lysates were used for quantification of SOD and MDA using commercially available kits (Solarbio, China).

### Construction of a myocardial ischemia‒reperfusion injury model in mice

The anterior descending branch of the left coronary artery (LAD) was ligated after the mice were anesthetized with 2% isoflurane. A 5-0 Prolene suture was placed at approximately 2 cm around the root of the left anterior descending coronary artery and released 30 min later to allow myocardial ischemia followed by reperfusion. Buprenorphine hydrochloride, as an analgesic (0.05 mg/kg), was given subcutaneously intraoperatively.

### Statistical analysis

Each of the above experiments was performed at least three times. The experimental data were statistically analyzed using GraphPad Prism 8.0, and the results are presented as the mean ± SD. p < 0.05 was considered statistically significant. Student’s t test was used to compare the differences between the samples, and one-way ANOVA was used to evaluate the differences among multiple samples.

## Results

### I/R damage cell model establishment and detection

**Fig. 1:**
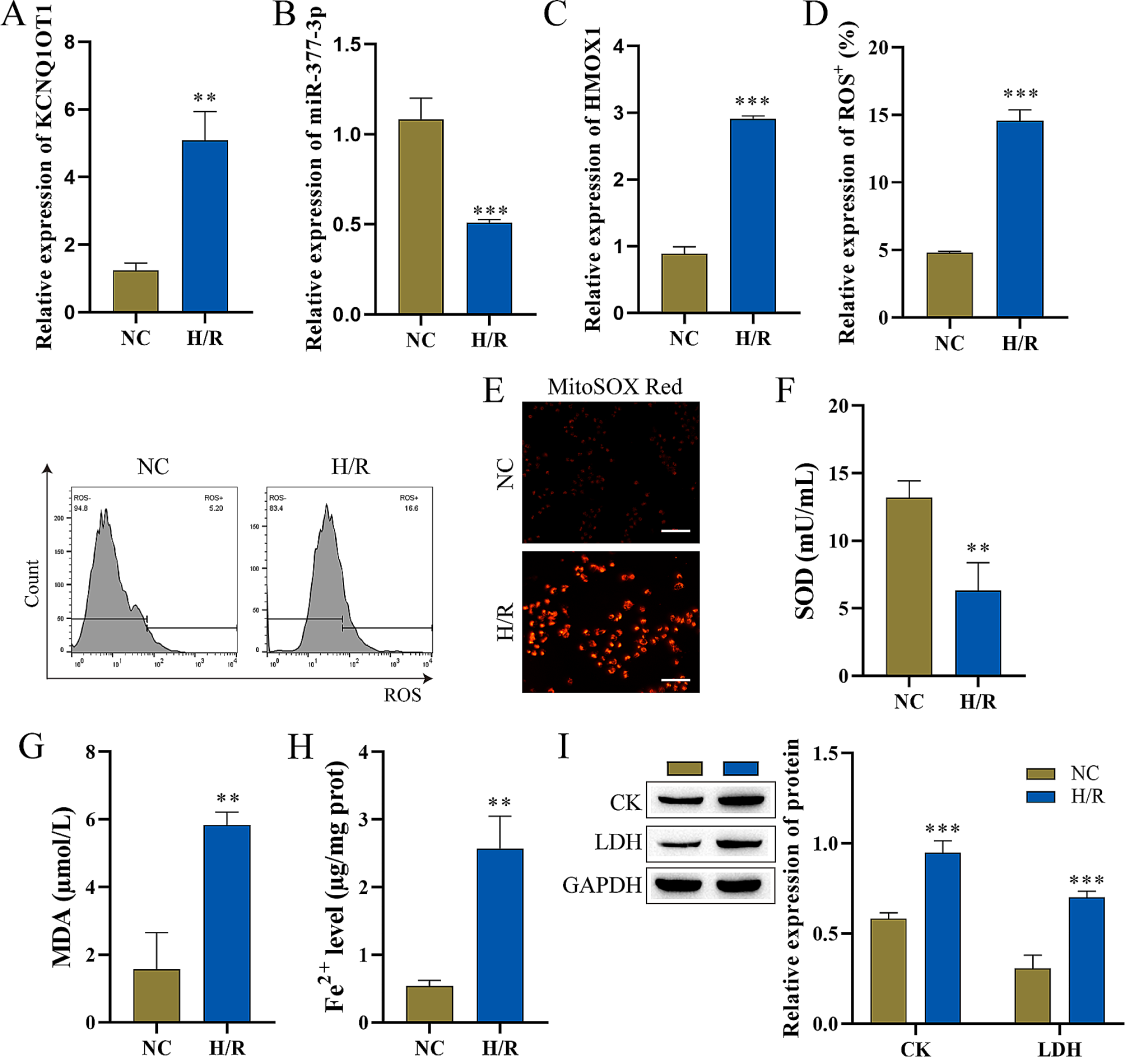
The establishment and detection of I/R damage cell model (n = 3). **A**-**C**: Detection of the expression of lncRNA KCNQ1OT1, miR-377-3p and HMOX1 by RT‒qPCR. D: Flow cytometry was used to detect ROS levels. E: MitoSOX Red probe was used to measure mitochondrial ROS levels (scale bar = 100 μm). **F**-**G**: Oxidative stress marker kits were utilized to evaluate SOD and MDA levels. **H**: Iron analysis kits were used to measure Fe^2+^ levels. I: CK and LDH expression levels were determined by western bolt. Compared with the NC group, ^**^ P < 0.01 and ^***^P < 0.001

First, we constructed an I/R injury cell model using HACMs. RT‒qPCR was used to detect the levels of lncRNA KCNQ1OT1, miR-377-3p, and HMOX1. The constructed I/R injury cell model had high levels of lncRNA KCNQ1OT1 and HMOX1 but miR-377-3p (Fig. 1A-C). Higher levels of ROS were detected in the I/R injury cell model than in the control (Fig. 1D). MitoSOX Red probe was used to measure mitochondrial ROS levels, the increased mitochondrial ROS levels was observed in H/R group (Fig. 1E). The levels of SOD and MDA were detected using oxidative stress marker assay kits, which demonstrated that the I/R injury cell model expressed low levels of SOD but high levels of MDA (Fig. 1F-G). Next, changes in Fe^2+^ were assayed using the iron assay kit, and the results showed significantly higher levels of Fe^2+^ in the I/R injury cell model (Fig. 1H). In addition, Western blot analysis demonstrated high levels of the myocardial injury markers (CK and LDH) in the I/R injury cells (Fig. 1I). These results indicated the successful establishment of a cardiomyocyte I/R injury model. LncRNA KCNQ1OT1, HMOX1 and miR-377-3p were expressed abnormally in I/R cell model.

### Effect of LncRNA KCNQ1OT1 on MI/RI

**Fig. 2:**
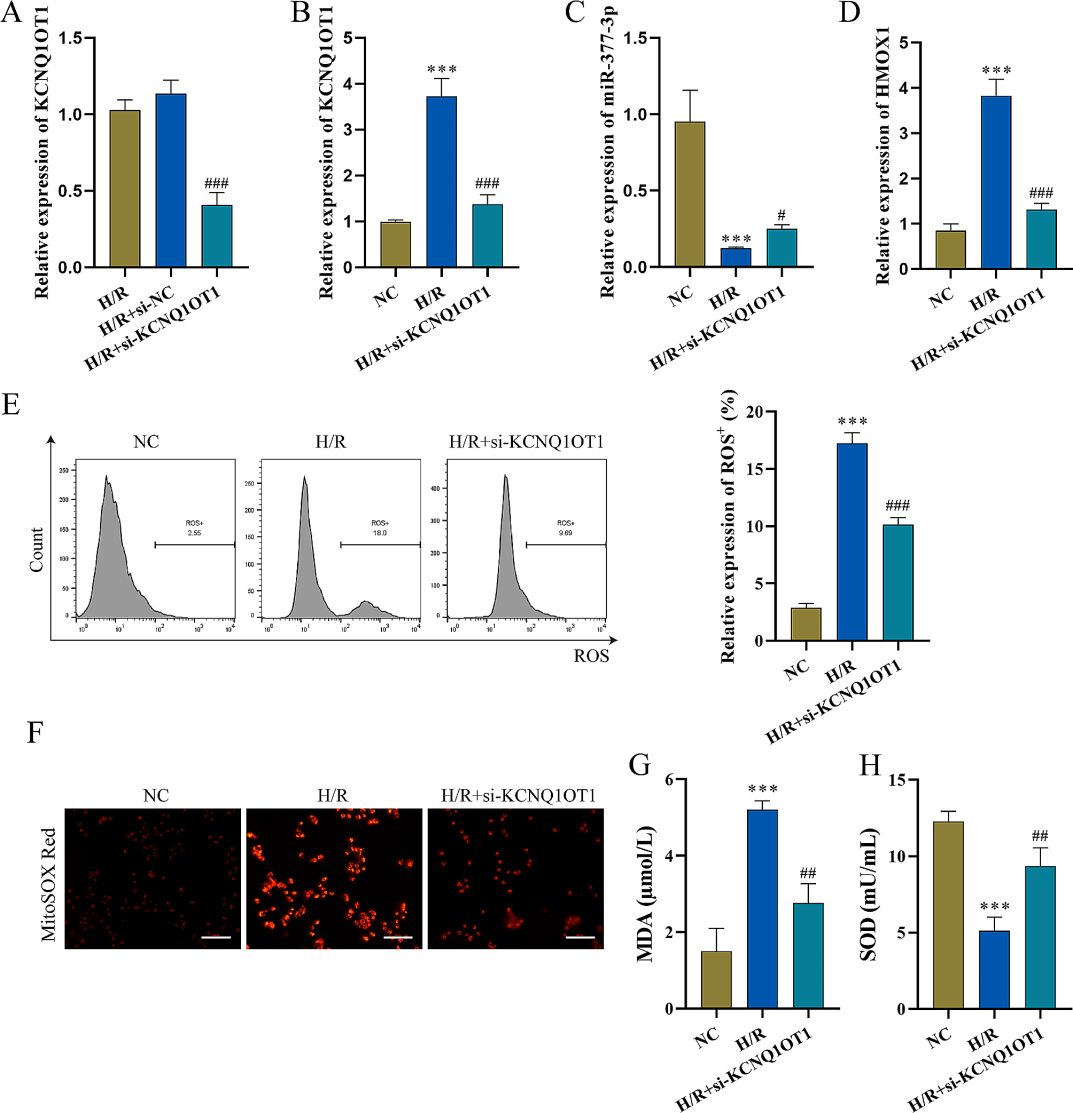
Effect of lncRNA KCNQ1OT1 on MI/RI (n = 3). **A**-**D**: Detection of the level of lncRNA-KCNQ1OT1, miR-377-3p, and HMOX1 by RT‒qPCR. E: Flow cytometry was used to detect ROS levels. **F**: MitoSOX Red probe was used to measure mitochondrial ROS levels (scale bar = 100 μm). **G**-**H**: Oxidative stress marker kits were utilized to evaluate SOD and MDA levels. Compared with the NC group, ^***^P < 0.001; compared with the H/R+si-NC or H/R + si-KCNQ1OT1 group, ^**#**^ P < 0.05, ^**##**^ P < 0.01 and ^###^ P < 0.001

To confirm the biological influence of lncRNA-KCNQ1OT1 in MI/RI injury, a small interfering RNA (siRNA) targeting lncRNA-KCNQ1OT1 was used, and RT‒qPCR demonstrated high transfection efficiency of the siRNA (Fig. 2A). After silencing lncRNA-KCNQ1OT1, the expression of lncRNA-KCNQ1OT1 and HMOX1 was significantly decreased, and miR-377-3p was increased in comparison to the H/R group (Fig. 2B-D). Intracellular ROS levels were significantly reduced under si-KCNQ1OT1 (Fig. 2E). MitoSOX Red probe was used to measure mitochondrial ROS levels, si-KCNQ1OT1 pretreatment attenuated the increase in H/R-induced mitochondrial ROS levels (Fig. 2F). Compared with the H/R group, the MDA content was significantly reduced, but the SOD content was increased in the H/R + si-KCNQ1OT1 group (Fig. 2G-H). These results suggested that knockdowning lncRNA KCNQ1OT1 alleviates MI/RI.

### Dual-luciferase reporter gene validation of the targeting action of lncRNA-KCNQ1OT1 in relation to miR-377-3p

**Fig. 3:**
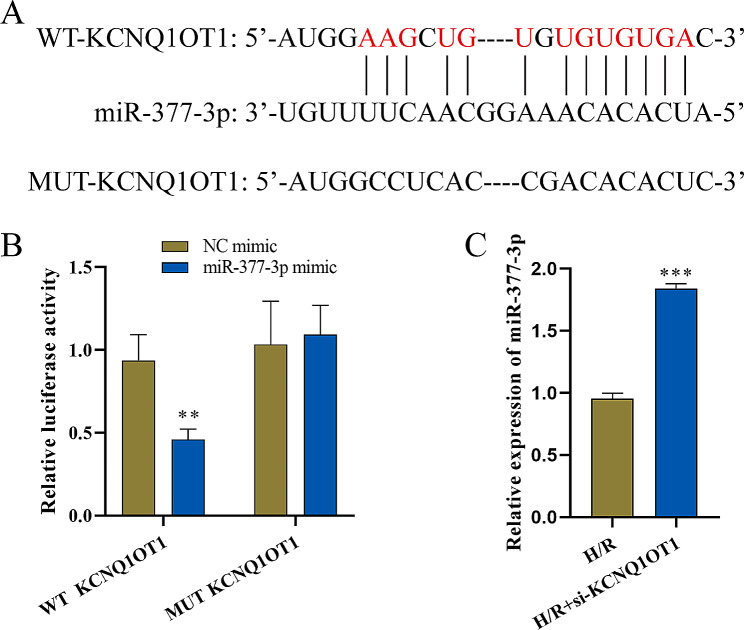
Targeting relationship between lncRNA KCNQ1OT1 and miR-377-3p (n = 3). **A**: Prediction of the binding sites between lncRNA KCNQ1OT1 and miR-377-3p by Starbase. **B**: Dual-luciferase validation of the KCNQ1OT1 3′UTR bound by miR-377-3p. **C**: RT‒qPCR detection of miR-377-3p expression. Compared with the NC mimic or H/R group, ^**^ P < 0.01 and ^***^ P < 0.001

First, Starbase predicted the target binding site between lncRNA KCNQ1OT1 and miR-377-3p (Fig. 3A). A dual-luciferase reporter gene assay demonstrated that the miR-377-3p mimic significantly reduced the luciferase activity of the KCNQ1OT1 wild‐type (WT) reporter but had no impact on the luciferase activity of the KCNQ1OT1 mutant (MUT) reporter (Fig. 3B). Subsequently, HACMs were transfected with si-KCNQ1OT1, and RT‒qPCR was used to detect the influence of lncRNA-KCNQ1OT1 on miR-377-3p. miR-377-3p was significantly increased after knockdown of KCNQ10T1 (Fig. 3C), which indicates that lncRNA-KCNQ1OT1 negatively regulates miR-377-3p.

### Influence of mir-377-3p on MI/RI

**Fig. 4:**
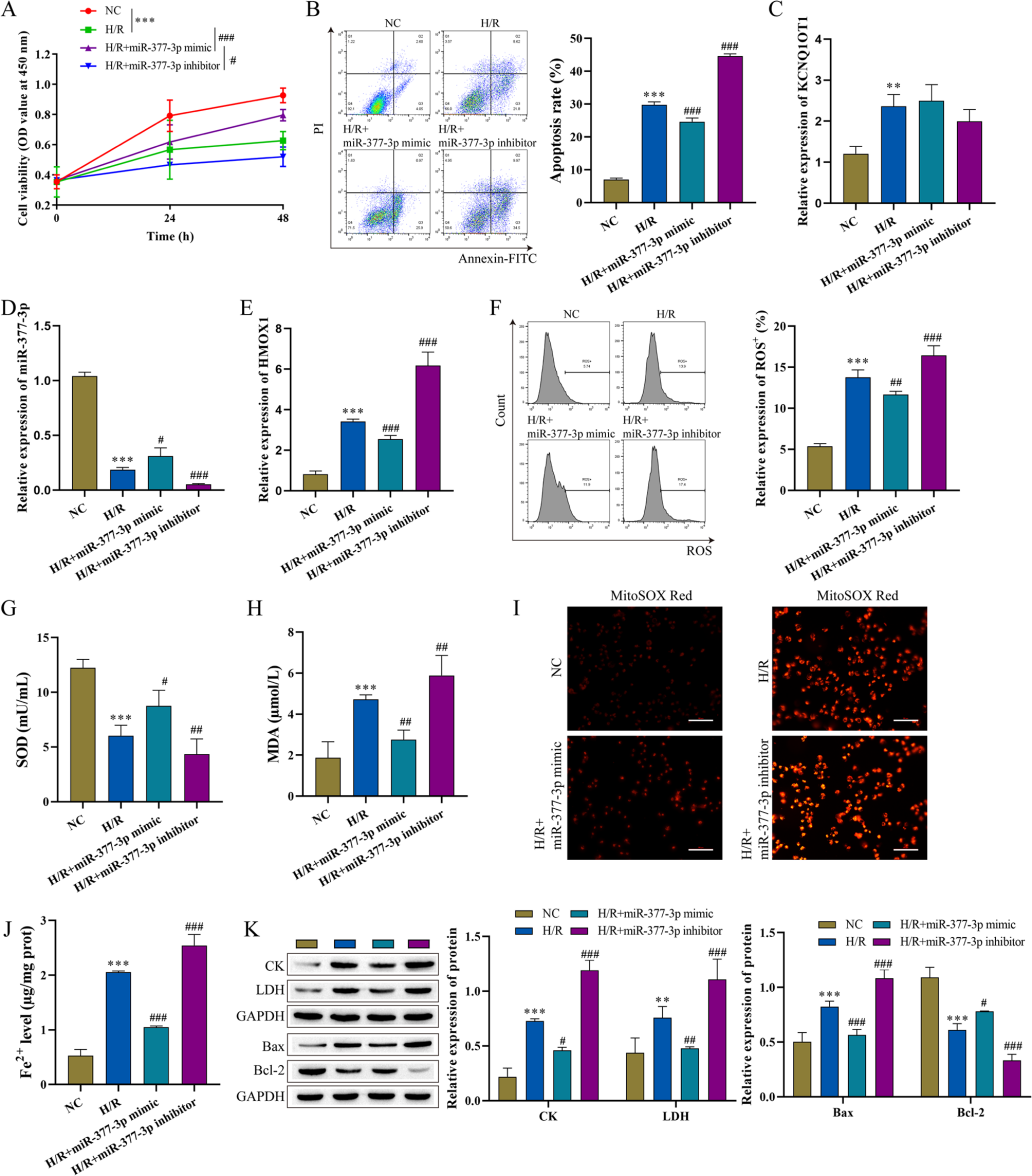
Effect of miR-377-3p on MI/RI (n = 3). **A**: CCK-8 assay was used to detect cell viability. **B**: Detection of cell apoptosis by flow cytometry. **C**-**E**: Detection of the level of lncRNA-KCNQ1OT1, miR-377-3p, and HMOX1 by RT‒qPCR. **F**: Flow cytometry analysis of ROS levels. **G**-**H**: Oxidative stress marker kits were used to measure SOD and MDA levels. **I**: MitoSOX Red probe was used to measure mitochondrial ROS levels (scale bar = 100 μm). **J**: Iron analysis kits were used to measure Fe^2+^ levels. **K**: CK, LDH, Bax and Bcl-2 expression levels were determined by western bolt. Compared with the NC group, ^**^ P < 0.01, and ^***^ P < 0.001; compared with the H/R group, ^**#**^ P < 0.05, ^**##**^ P < 0.01 and ^###^ P < 0.001

To confirm the specific role of miR-377-3p in HACMs, miR-377-3p was overexpressed or knocked down. After addition of miR-377-3p mimic, cell viability of HACMs was significantly increased, while miR-377-3p inhibitor significantly decreased the cell viability (Fig. 4A). In the miR-377-3p mimic group, the apoptosis of HACMs was decreased, but the apoptosis was increased after miR-377-3p inhibition (Fig. 4B). Overexpression of miR-377-3p in HCAMs resulted in increased levels of miR-377-3p, but decreased levels of HMOX1. In contrast, inhibition of miR-377-3p resulted in decreased expression of miR-377-3p, but increased HMOX1 (Fig. 4C-E). Flow cytometry analysis demonstrated a significant decrease in ROS content in the miR-377-3p mimic group but an increase in the miR-377-3p inhibitor group (Fig. 4F). MitoSOX Red analysis showed similar results (Fig. 4I). In the miR-377-3p mimic group, the content of SOD was increased, but MDA was decreased. However, the contents of SOD and MDA were reversed in the miR-377-3p inhibitor group (Fig. 4G-H). Meanwhile, the content of Fe^2+^ was significantly decreased after miR-377-3p overexpression, while Fe^2+^ was increased after miR-377-3p inhibition (Fig. 4J). Western blot showed miR-377-3p promoted the expression of Bcl-2 but inhibited the expression of Bax, CK, LDH (Fig. 4K). These results indicated that miR-377-3p inhibited I/R-induced cell apoptosis and oxidative stress in HACMs.

### Dual-luciferase reporter gene assay validation of the targeting relationship between mir-377-3p and HMOX1

**Fig. 5:**
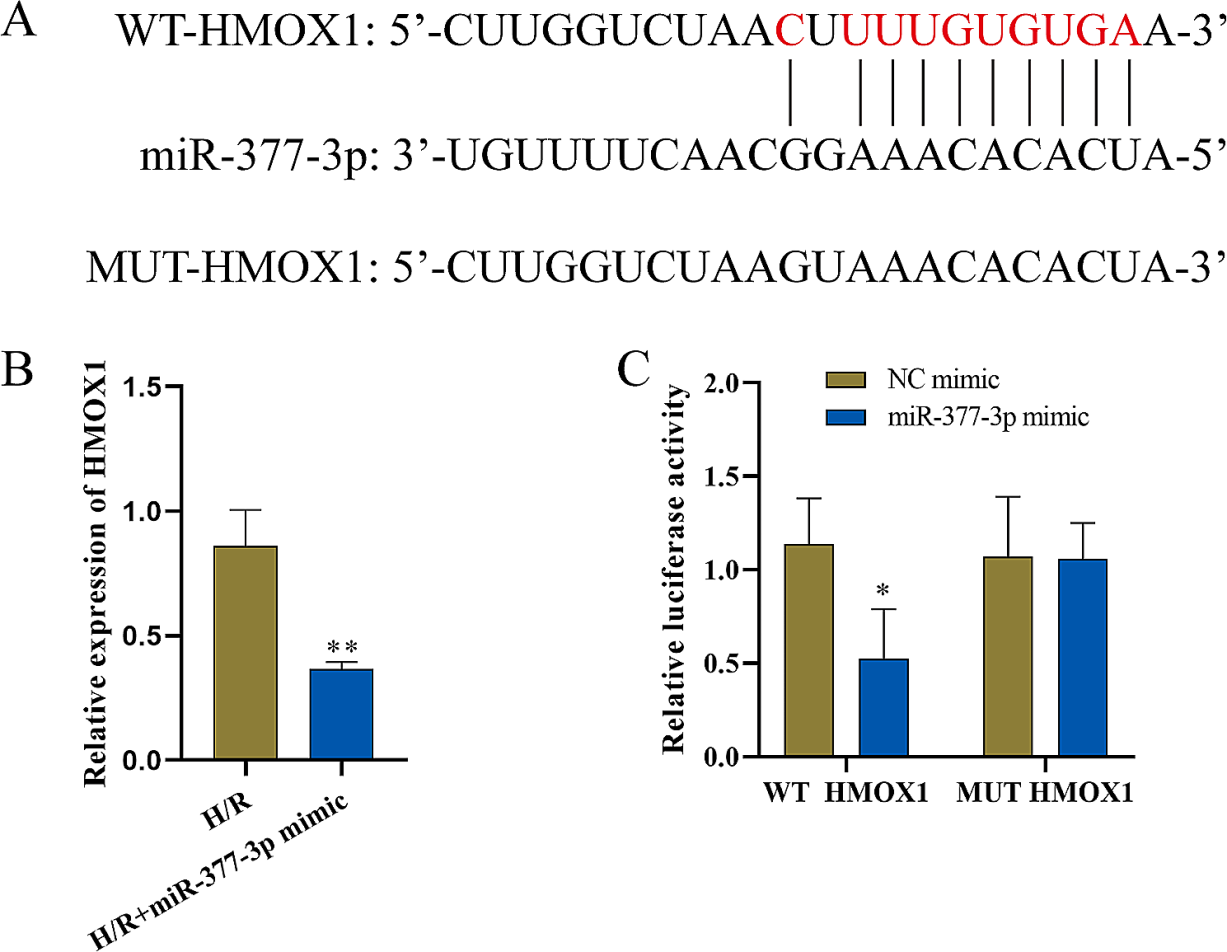
Targeting relationship between miR-377-3p and HMOX1 (n = 3). **A**: Starbase was used to predict the binding site of miR-377-3p to HMOX1. **B**: The level of HMOX1 was detected by RT‒qPCR. **C**: A dual-luciferase reporter assay was used to detect the binding relationship of miR-377-3p and the HMOX1 3′UTR. Compared with the NC mimic or H/R group, ^*^ P < 0.05 and ^**^ P < 0.01

First, Starbase predicted the target binding site between miR-377-3p and HMOX1 (Fig. 5A). Next, RT‒qPCR was performed to detect the level of HMOX1. After miR-377-3p overexpression, the level of HMOX1 was significantly decreased (Fig. 5B). In addition, a dual-luciferase reporter gene assay demonstrated that the miR-377-3p mimic significantly reduced the luciferase activity of the HMOX1 WT reporter but had no impact on the luciferase activity of the HMOX1 MUT reporter (Fig. 5C). These results indicated that miR-377-3p targeted HMOX1 and had a negative regulatory effect.

### Inhibition of HMOX1 attenuates mir-377-3p downregulation-induced myocardial injury

**Fig. 6:**
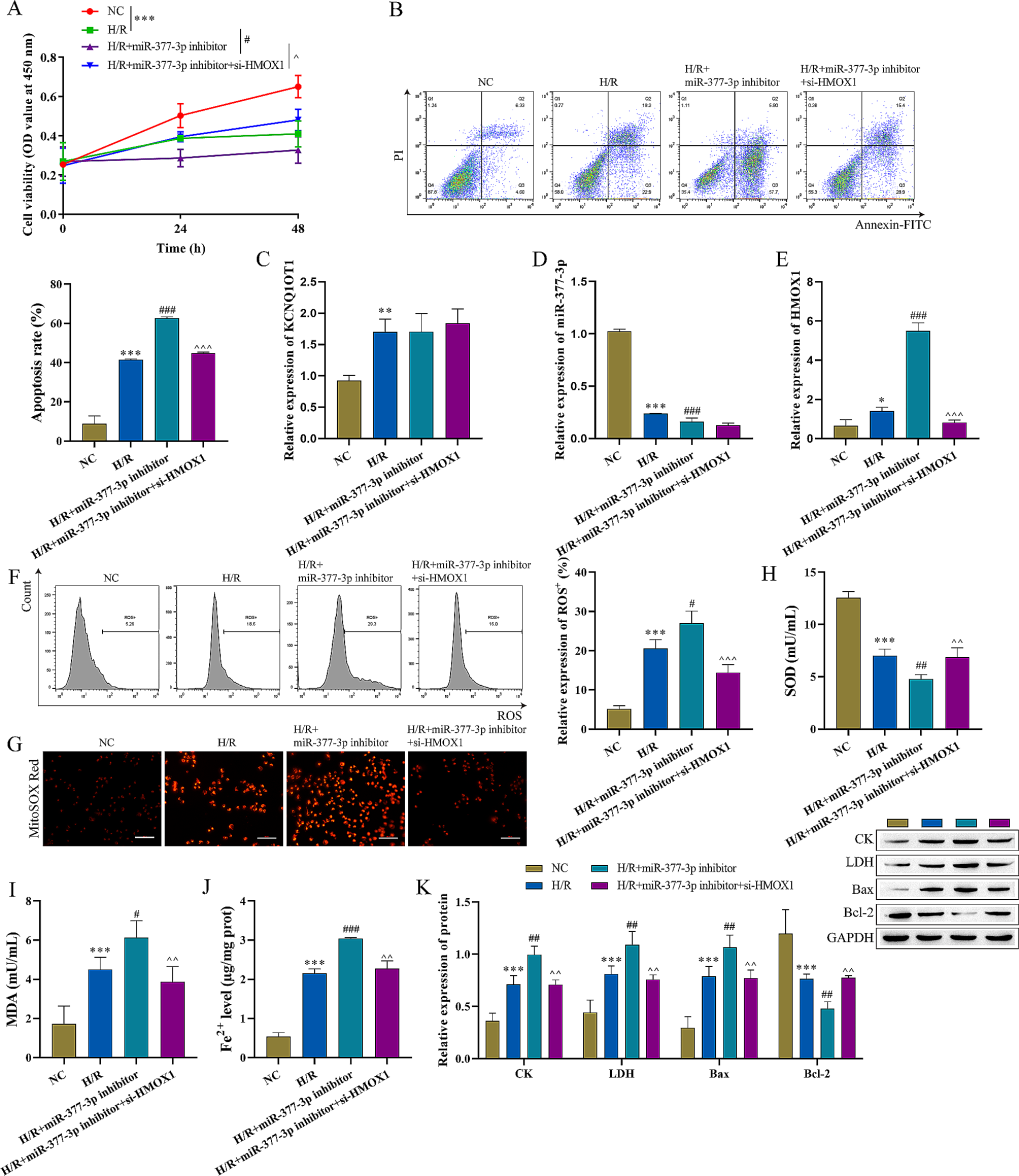
Inhibition of HMOX1 attenuates miR-377-3p downregulation-induced myocardial injury (n = 3). A: CCK-8 assay was used to detect cell viability of HACMs. B: Flow cytometry was used to detect cell apoptosis. C-E: RT‒qPCR was used to measure the expression of miR-377-3p, HMOX1, RNA KCNQ1OT1. F: Flow cytometry was used to detect ROS levels. G: MitoSOX Red probe was used to measure mitochondrial ROS levels (scale bar = 100 μm). H-I: Oxidative stress marker kits were used to detect the expression of SOD and MDA. J: Iron analysis kit was utilized to detect changes in Fe^2+^. K: Western blot analysis was used to detecting the expression of CK, LDH, Bax, and Bcl-2. Compared with the NC group, ^*^ P < 0.05, ^**^ P < 0.01 and ^***^ P < 0.001; compared to with H/R group, ^**#**^ P < 0.05, ^**##**^ P < 0.01 and ^###^ P < 0.001; compared with the H/R + miR-377-3p inhibitor group, ^^^P < 0.05, ^^^^P < 0.01 and ^^^^^P < 0.001

We further investigated the influence of HMOX1 inhibition on MI/RI and ferroptosis. HMOX1 inhibition was used in HACMs. Compared to the H/R + miR-377-3p inhibitor group, cell viability of HACMs was improved and the apoptosis was decreased in H/R + miR-377-3p-inhibitor + si-HMOX1 group (Fig. 6A-B). RT-qPCR assay showed that compared with the H/R + miR-377-3p inhibitor group, both HMOX1 and miR-377-3p were decreased, but the expression of RNA KCNQ1OT1 was increased in the H/R + miR-377-3p inhibitor + si-HMOX1 group (Fig. 6C-E). Flow cytometry and MitoSOX Red demonstrated a significant decrease in intracellular ROS in H/R + miR-377-3p inhibitor + si-HMOX1 (Fig. 6F-G). Compared with the H/R + miR-377-3p inhibitor group, the intracellular SOD content was increased and the MDA was decreased subjected to HMOX1 inhibition (Fig. 6H-I), which indicated that HMOX1 inhibition inhibited oxidative stress. Fe^2+^ was detected by an iron analysis kit, which demonstrated that the Fe^2+^ content was decreased in HACMs after HMOX1 inhibition (Fig. 6J). Finally, we detected the levels of CK, LDH, Bax, and Bcl-2. Compared with the H/R + miR-377-3p-inhibitor group, CK, LDH and Bax were significantly decreased, while Bcl-2 expression was significantly increased in the H/R + miR-377-3p inhibitor + si-HMOX1 group (Fig. 6K). These results indicated that HMOX1 inhibition alleviates miR-377-3p downregulation-induced MI/RI and inhibits ferroptosis.

### LncRNA KCNQ1OT1 targets mir-377-3p to mediate I/R via the HMOX1 pathway

**Fig. 7:**
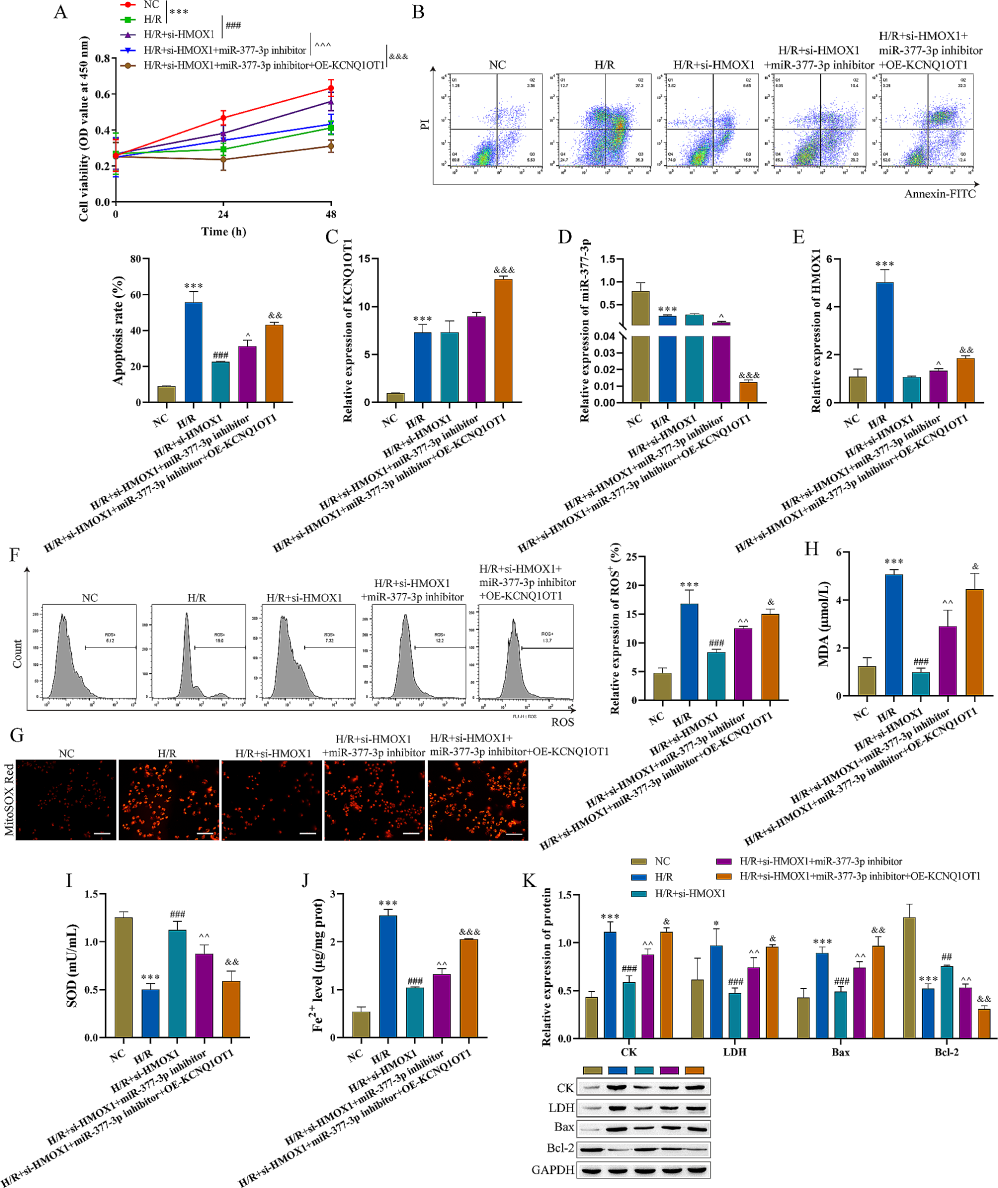
LncRNA KCNQ1OT1 targets miR-377-3p to mediate I/R injury via the HMOX1 pathway (n = 3). A: CCK-8 assay was used to detect the proliferation of HACMs. B: Flow cytometry was used to detect HACMs apoptosis. C-E: RT‒qPCR was used to detect the expression of lncRNA KCNQ1OT1, miR-377-3p, and HMOX1. F: Flow cytometry was used to measure ROS levels. G: MitoSOX Red probe was used to measure mitochondrial ROS levels (scale bar = 100 μm). H-I: Oxidative stress marker kits were utilized to measure SOD and MDA expression. J: An iron analysis kit was used to detect changes in Fe^2+^. K: Western blot analysis was used to detect the levels of CK, LDH, Bax, and Bcl-2. Compared with the NC group, ^*^ P < 0.05, ^**^ P < 0.01 and ^***^ P < 0.001; compared with the H/R group, ^**#**^ P < 0.05, ^**##**^ P < 0.01 and ^**###**^ P < 0.001; compared with the H/R + si-HMOX1 group, ^P < 0.05, ^^P < 0.01 and ^^^P < 0.001; compared with the H/R + si-HMOX1 + miR-377-3p inhibitor group, ^&^P < 0.05, ^&&^ P < 0.01 and ^&&&^ P < 0.001

First, lncRNA KCNQ1OT1 was overexpressed in H/R + si-HMOX1 + miR-377-3p inhibitor group, resulted in attenuation of cell viability and increase of cell apoptosis (Fig. 7A-B). Next, The RT‒qPCR results showed that compared with H/R + si-HMOX1 + miR-377-3p inhibitor group, lncRNA KCNQ1OT1 and HMOX1 increased, while the level of miR-377-3p was significantly decreased in H/R + si-HMOX1 + miR-377-3p inhibitor + OE-KCNQ1OT1 group (Fig. 7C-E). An increase in intracellular ROS in HACMs was detected by flow cytometry after lncRNA KCNQ1OT1 overexpression (Fig. 7F). MitoSOX Red probe was used to measure mitochondrial ROS levels, OE-KCNQ1OT1 pretreatment promoted the increase in H/R + si-HMOX1 + miR-377-3p -induced mitochondrial ROS levels (Fig. 7G). SOD content was decreased but that MDA was increased after lncRNA KCNQ1OT1 overexpression in comparison to H/R + si-HMOX1 + miR-377-3p inhibitor group (Fig. 7H-I), which indicated that lncRNA KCNQ1OT1 promoted oxidative stress by miR-377-3p/HMOX1. The content of Fe^2+^ was increased in HACMs after lncRNA KCNQ1OT1 overexpression (Fig. 7J). Finally, compared with the H/R + miR-377-3p inhibitor + si-HMOX1 group, the intracellular levels of CK, LDH, and Bax were significantly increased, but the Bcl-2 levels were decreased in H/R + si-HMOX1 + miR-377-3p inhibitor + OE-KCNQ1OT1 group (Fig. 7K). These results indicated that lncRNA KCNQ1OT1 targets miR-377-3p to mediate I/R injury via the HMOX1 pathway.

### Animal experimental validation of the influence of lncRNA KCNQ1OT1 on MI/RI via the miR-377-3p/HMOX1 pathway

**Fig. 8:**
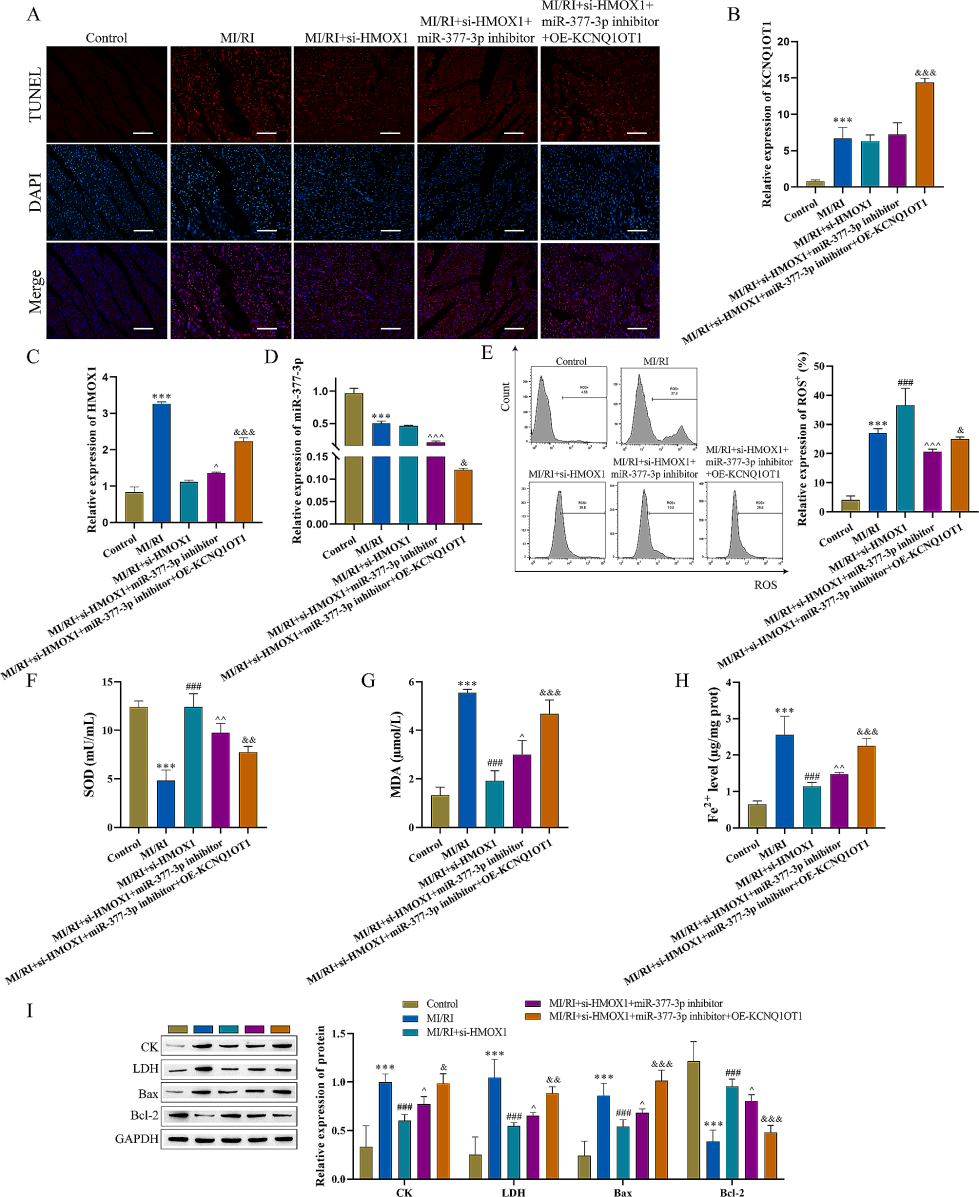
Animal experimental validation of the influence of lncRNA KCNQ1OT1 on MI/RI via the miR-377-3p/HMOX1 pathway (n = 5). A: Apoptosis was measured by a TUNEL assay (scale bar = 100 μm). B-D: RT‒qPCR was used to measure lncRNA KCNQ1OT1, miR-377-3p, and HMOX1 expression. E: An ROS assay kit was utilized to measure ROS levels. F and G: Oxidative stress marker kits were used to evaluate SOD and MDA expression. H: An iron analysis kit was used to detect Fe^2+^ changes. I: Western blot analysis was used to detect the levels of CK, LDH, Bax, and Bcl-2. Compared with the control group, ^*^ P < 0.05, ^**^ P < 0.01 and ^***^ P < 0.001; compared with the MI/RI group, ^**#**^ P < 0.05, ^**##**^ P < 0.01 and ^**###**^ P < 0.001; compared with the MI/RI + si-HMOX1 group, ^P < 0.05, ^^P < 0.01 and ^^^P < 0.001; compared with the MI/RI + si-HMOX1 + miR-377-3p inhibitor group, ^&^P < 0.05, ^&&^ P < 0.01 and ^&&&^ P < 0.001

To further verify the relationship among the effects of lncRNA KCNQ1OT1, miR-377-3p, and HMOX1, experiments were performed using a MI/RI mouse model. The apoptosis rate of atrial tissue cells was significantly decreased after knockdowning HMOX1, miR-388-3p inhibitor promoted the apoptosis and lncRNA KCNQ1OT1 further promoted the apoptosis (Fig. 8A). RT‒qPCR analysis demonstrated that the levels of lncRNA KCNQ1OT1 and HMOX1 were increased, but that the levels of miR-377-3p were decreased in atrial tissue with lncRNA KCNQ1OT1 overexpression (Fig. 8B-D). After MI/RI, an increase in ROS and MDA, but a decrease in SOD. ROS and MDA were inhibited after knockdowning HMOX1, miR-388-3p inhibitor promoted the level of ROS and MDA, but inhibited the level of SOD. lncRNA KCNQ1OT1 further promoted the level of ROS and MDA, but inhibited SOD (Fig. 8E-G). The content of Fe^2+^ in HACMs was significantly increased after lncRNA KCNQ1OT1 overexpression (Fig. 8H). In addition, compared with the MI/RI + si-HMOX1 + miR-377-3p inhibitor group, CK, LDH and Bax were significantly increased, while Bcl-2 expression was significantly decreased in atrial tissue after overexpressing lncRNA KCNQ1OT1 (Fig. 8I). The in vitro experiments were consistent with the results described above, further validates the mechanism of the lncRNA KCNQ1OT1/miR-377-3p/HMOX1 ceRNA network in MI/RI.

## Discussion

MI/RI leads to apoptosis and cytonecrosis of cardiomyocytes, and it may even lead to cardiac arrest [2]. The mechanisms of MI/RI are complex and involve reperfusion exacerbating cellular ROS production, increasing oxidative stress, and activating downstream transcription factors to aggravate inflammation and accelerate cell death [24, 25]. However, the specific molecular mechanisms of action are not fully understood. An important regulatory effect of the lncRNA‒miRNA-mRNA ceRNA network in MI/RI has been reported [26–28]. Yingping Liang et al. [29] found that the inflammatory response induced by ischemia‒reperfusion injury of human cardiomyocytes is regulated by the lncRNA ROR/miR-124-3p/TRAF6 axis. Xueying Tong et al. [30] reported that MI/RI is regulated by lncRNA LSINCT5/miR-222 through the PI3K/AKT pathway. Zhihao Guo et al. [31] also found that MI/RI is alleviated by mitochondrial apoptosis mediated by miR-503-5p/BIRC5, and they reported that this apoptosis is inhibited by lncRNA PART1. The present study found that the lncRNA KCNQ1OT1/miR-377-3p/HMOX1 ceRNA network has important regulatory roles in MI/RI, and a series of experiments were performed to confirm the specific molecular mechanisms of action and the relationships among their actions.

LncRNAs and miRNAs both have been shown to influence I/R injury. LncRNA KCNQ1OT1 has been reported to promote cardiomyocyte injury by inducing apoptosis, inhibiting cell proliferation, and exacerbating oxidative stress [14]. We constructed a siRNA against lncRNA KCNQ1OT1 and detected a decrease in intracellular ROS levels, an increase in SOD levels, and a significant decrease in MDA levels in the si-KCNQ1OT1 group, which further supported the previously reported findings. In previous studies, many miRNAs have been found to act as key regulators of cardiomyocyte activity, proliferation, and apoptosis [32, 33]. For example, miR-125b reduces myocardial infarct size and inhibits myocardial ischemia‒reperfusion injury [34], and miRNA-30c-5p prevents MI/RI by regulating Bach1/Nrf2 [35]. In the present study, we found that miR-377-3p was expressed at low levels in the H/R cell model. The miR-377-3p inhibitor significantly increased the contents of ROS and MDA but significantly decreased the contents of SOD compared to the H/R group. Western blot analysis demonstrated an increase in the expression of myocardial injury markers (LDH and CK), a significant decrease in Bcl-2, and an increase in Bax after treatment with miR-377-3p inhibitor. In addition, Fe^2+^ levels were significantly increased after miR-377-3p inhibition. Moreover, all of these changes were opposite after overexpression of miR-377-3p. These results confirmed the regulatory effect of miR-377-3p on cardiomyocytes by downregulating the promotion of oxidative stress and MI/RI in HACMs.

It has been reported that lncRNA-targeted miRNAs regulate MI/RI damage, and Yinghao Pei et al. [36] found that lncRNA PEAMIR acts as a competitive endogenetic RNA for miR-29b-3p, which is aggravated by apoptosis and inflammatory responses in MI/RI. Shuang Wang et al. [37] reported that lncRNA MALAT1 induces cardiomyocyte injury via miR-20b. In the present study, we used a dual-luciferase assay to verify the relationship between lncRNA KCNQ1OT1 and miR-377-3p. Compared to the H/R + si-HOMX1 + miR-377-3p inhibitor group, overexpression of KCNQ1OT1 induced cell apoptosis, increased intracellular levels of Fe^2+^, ROS and MDA, decreased SOD levels, and increased the expression of myocardial injury markers (CK and LDH).

Heme oxygenase 1 (HMOX1), as a cell protective enzyme, degrades heme [38]. HMOX1 activity provides antioxidant, antiapoptotic, and cytoprotective effects through its catabolic metabolites [39]. HMOX1 activation mediates the release of free iron ions from heme, and iron plays a key role in the formation and destruction of ROS [6, 7]. HMOX1 inhibits oxidative stress [40], but the accumulation of excess iron ions in cardiomyocytes tends to induce iron death [41]. Therefore, exploring the molecular mechanisms by which HMOX1 regulates iron and oxidative stress in MI/RI is of great importance for the treatment of heart-related diseases. In the present study, we demonstrated that miR-377-3p targets HMOX1 using a dual-luciferase reporter assay. Compared to the H/R + miR-377-3p inhibitor group, knockdown of HMOX1 inhibited cell apoptosis, decreased intracellular levels of Fe^2+^, ROS and MDA, increased SOD levels, and decreased the expression of myocardial injury markers (CK and LDH).

Finally, animal experiments demonstrated that the miR-377-3p inhibitor reverses the protective effect of HOMX knockdown on MI/RI. Overexpression of lncRNA KCNQ1OT1 further exacerbates MI/RI, including apoptosis, as well as increases the level of oxidative stress and the expression of CK and LDH. The present study provided potential therapeutic strategies for better elucidating the mechanism, clinical prevention, and treatment of MI/RI, thereby laying the experimental foundation for future studies.

## Data Availability

The datasets used and/or analyzed during the current study are available from the corresponding author upon reasonable request.
